# N6-Methyladenosine-Related Gene Signature Associated With Monocyte Infiltration Is Clinically Significant in Gestational Diabetes Mellitus

**DOI:** 10.3389/fendo.2022.853857

**Published:** 2022-03-18

**Authors:** Runyu Du, Ling Li, Yanjun Wang

**Affiliations:** Department of Endocrinology, Shengjing Hospital of China Medical University, Shenyang, China

**Keywords:** gestational diabetes mellitus, N6-methyladenosine modification, immune infiltration, monocyte, nomogram

## Abstract

**Objective:**

The objective of this study was to reveal the potential crosstalk between immune infiltration and N^6^- methyladenosine (m^6^A) modification in the placentas of patients with gestational diabetes mellitus (GDM), and to construct a model for the diagnosis of GDM.

**Methods:**

We analyzed imbalanced immune infiltration and differentially expressed m^6^A-related genes (DMRGs) in the placentas of patients with GDM, based on the GSE70493 dataset. An immune-related DMRG signature, with significant classifying power and diagnostic value, was identified using a least absolute shrinkage and selection operator (LASSO) regression. Based on the selected DMRGs, we developed and validated a nomogram model using GSE70493 and GSE92772 as the training and validation sets, respectively.

**Results:**

Infiltration of monocytes was higher in GDM placentas than in control samples, while the infiltration of macrophages (M1 and M2) in GDM placentas was lower than in controls. A total of 14 DMRGs were strongly associated with monocyte infiltration, seven of which were significant in distinguishing patients with GDM from normal controls. These genes were *CD81*, *CFH*, *FABP5*, *GBP1*, *GNG11*, *IL1RL1*, and *SLAMF6*. The calibration curve, decision curve, clinical impact curve, and receiver operating characteristic curve showed that the nomogram recognized GDM with high accuracy in both the training and validation sets.

**Conclusions:**

Our results provide clues that crosstalk between m^6^A modification and immune infiltration may have implications in terms of novel biomarkers and therapeutic targets for GDM.

## Introduction

Gestational diabetes mellitus (GDM) is a form of diabetes that is first diagnosed during pregnancy, with a worldwide prevalence of 9–21%. GDM frequently affects both short-term and long-term health in the mother and offspring, because of the diverse genetic background and epigenetic modifications that occur in response to nutritional and environmental factors (1, 2). Currently, the precise etiological mechanisms of GDM remain unclear; however, numerous studies have found that GDM is a multifactorial disease that involves genetic factors, lifestyle, and chronic inflammation. Insulin resistance (IR) and pancreatic β-cell dysfunction are regarded as essential for the pathogenesis of GDM (2, 3). Although the exact mechanisms remain to be clarified, chronic inflammation has been reported to participate in the development of IR and pancreatic β cell failure, which in turn leads to GDM (4). The placenta is a temporary organ formed during pregnancy, which serves as the only bridge connecting the mother and fetus, and has important endocrine function. Placenta-derived inflammatory cytokines, such as interleukin-1 beta (IL-1β), IL-6, IL-15, IL-10, IL-34, IL-38, and tumor necrosis factor alpha (TNF-α), can stimulate immune cells and aggravate immune and inflammatory responses, thereby exacerbating chronic inflammation and maternal IR and inducing β cell failure during pregnancy (5–11). Moreover, immune cells and inflammatory cytokines are important components of the placental microenvironment, which is essential for normal pregnancy (12, 13). Imbalanced immune infiltration in the placenta contributes to the pathogenesis and development of pregnancy-specific diseases, including GDM, and may affect GDM-related adverse pregnancy outcomes and clinical prognosis (13–15).

Chemical modifications of cellular RNAs can result in secondary structure modifications, splicing, degradation, or molecular stability, which are emerging layers of post-transcriptional gene regulation. More than 160 chemical modifications have been identified (16). N^6^- methyladenosine (m^6^A) RNA modification is the most prevalent type of RNA epigenetic processing (17, 18). m6A modification is mediated by its effector proteinsare in a dynamic and reversible pattern (17). m6A occurs mainly in the 3’- UTR and the vicinity of the termination codon mRNA, which is recogonized by “readers” (YTH domain family (YTHDF]1–3, and insulin-like growth factor 2 mRNA-binding proteins 1–3), catalyzed by methylases [methyltransferase-like (METTL)3/14, and Wilms’ tumour 1-associated protein], and removed by demethylases [fat-mass and obesity-associated protein (FTO), and alkylation repair homolog protein 5] (19). Recent evidence indicates that perturbations of m^6^A modifications dysregulate mRNA metabolism, including mRNA stability, mRNA splicing, RNA nucleation, RNA-protein teractions and mRNA translation, thereby contributing to various physiological and pathophysiological processes (20–22). Numerous m^6^A modifications have been shown to regulate adipogenesis, glucose metabolism, insulin resistance, and the related chronic immune response (17, 21, 23). This suggests that m^6^A modifications are implicated in the development of metabolic diseases, although the specific knowledge regarding GDM is still in its infancy.

In this study, we aimed to reveal the imbalanced immune infiltration in the placenta of patients with GDM, the differentially expressed m^6^A-related genes (DMRGs) involved, as well as the crosstalk between them, and also to develop a nomogram model for the diagnosis of GDM.

## Materials and Methods

### Data Collection

**Figure 1:**
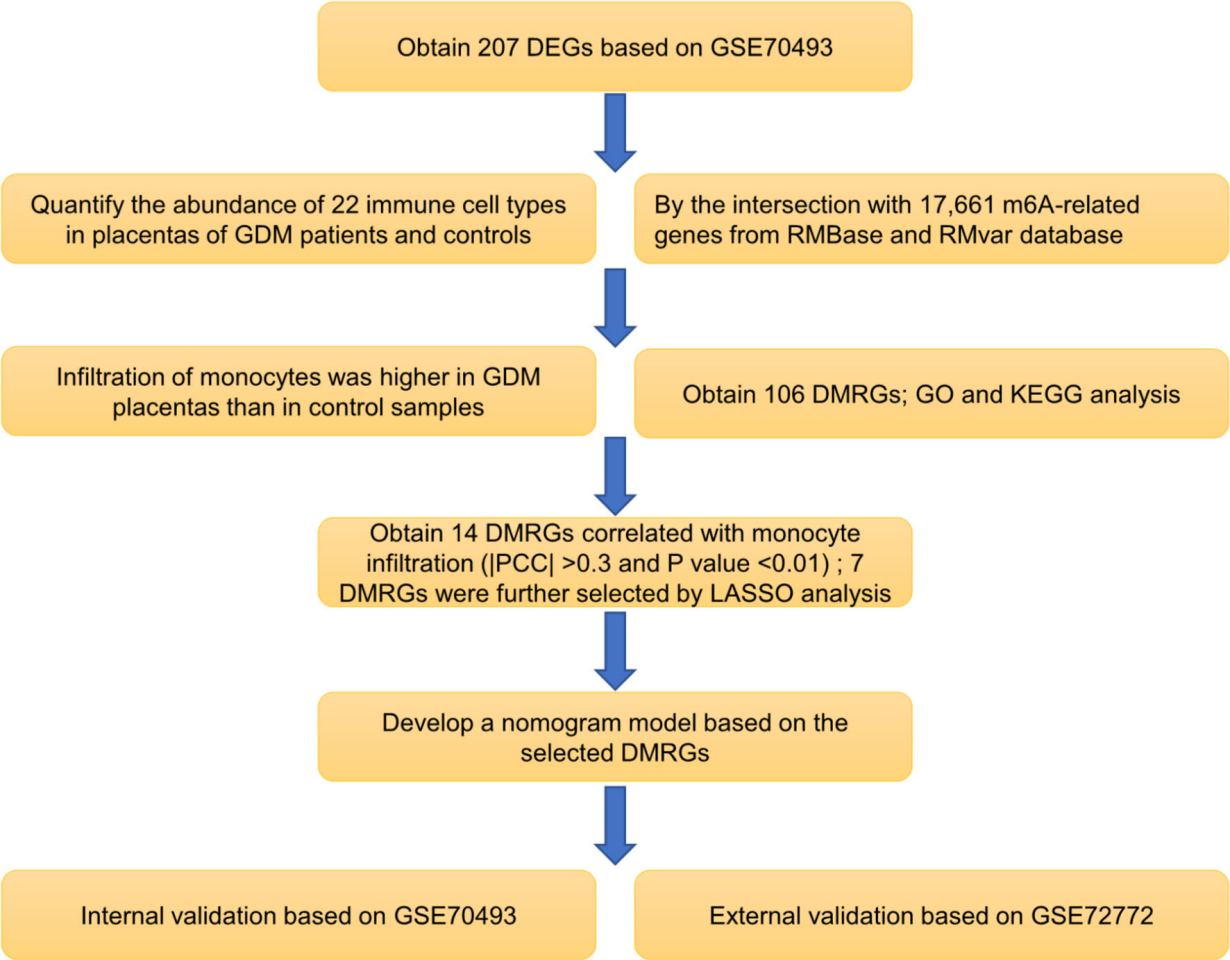
Flowchart of the research workflow. Abbreviations are defined as follows: differentially expressed gene (DEG), gestational diabetes mellitus (GDM), differentially expressed m6A-related gene (DMRG), Gene Ontology (GO), Kyoto Encyclopedia of Genes and Genomes (KEGG), Pearson correlation coefficient (PCC), least absolute shrinkage and selection operator (LASSO).

The human expression dataset GSE70493 was downloaded from the Gene Expression Omnibus database (https://www.ncbi.nlm.nih.gov/geo/). The expression data in GSE70493 contained 63 samples of the maternal placenta (GDM; n = 32 and normal glucose tolerance [NGT]; n = 31). We obtained 17,661 m^6^A-related genes by crossing data from the RMBase (24) and RMvar (25) databases. The expression data in GSE92772 were obtained for validation and contained RNA profiles of maternal whole blood cells from eight GDM and eight NGT pregnant women in their second trimester. The workflow of this research is shown in **Figure 1**.

### Screening of Differentially Expressed m^6^A-Related Genes

Using the R software ‘limma’ package, we identified differentially expressed genes (DEGs) in the GSE70493 dataset, based on the criteria of |log2 fold change| > 0.1 and *P* value < 0.01. Heatmaps were generated using the R software ‘pheatmap’ package. In the case of multiple probes corresponding to the same gene, we selected the probe with the lowest *P* value. Genes without official symbols were removed, and all symbols were converted to symbols approved by the HUGO Gene Nomenclature Committee. We then crossed the DEGs with m^6^A-related genes to obtain the DMRGs.

### Functional-Enrichment Analysis

To determine the potential functions and enriched pathways of DMRGs in GDM, Gene Ontology (GO) and Kyoto Encyclopedia of Genes and Genomes (KEGG) pathways were analyzed using the R software ‘enrichplot’ package. A *P* value of < 0.05 was set as the cutoff.

### Evaluation of Immune Cell Infiltration in Placenta

CIBERSORTx (26) was utilized to quantify the abundance of 22 immune cell types in each sample by imputing the gene expression profiles of GSE70493. We then compared the differences in immune cell infiltration between GDM patients and healthy subjects.

### Selection of Core DMRGs Correlated With Immune Infiltration

Pearson correlation coefficient (PCC) analysis was conducted to identify the DMRGs correlated with the differentially infiltrated immune cells between GDM and healthy patients. LASSO analysis, a linear regression model penalized with the L1 norm, was used to further narrow down the variables owing to its tendency to prefer solutions with fewer non-zero coefficients. A tuning parameter, lambda, was used to control the number of coefficients with a value of zero. The 10-fold K cross-validations for the centralization and normalization of selected variables to select the optimal lambda value using R software. The core DMRGs that correlated with immune infiltration were identified using LASSO analysis.

### Construction and Validation of the Nomogram Model for GDM Diagnosis

A logistic regression model was considered to evaluate the performance the core m6A-related genes selected by LASSO to estimate the probability of GDM. Based on this model, we constructed a nomogram for individual predictions of GDM using R software. To validate the classification ability of the nomogram model, calibration was analyzed using a bootstrapping approach and randomly repeated 1,000 times with replacement. Decision curve analysis and clinical impact curves were used to determine clinical usefulness. The receiver operating characteristic (ROC) curve was used to evaluate the sensitivity and specificity of the nomogram. To externally validate the nomogram, we then applied the calibration, decision curve, clinical impact curve, and ROC curve analysis on GSE92772.

### Statistical Analysis

Data processing and statistical analyses were performed using R software (version 4.0.3). Associations between quantitative variables were assessed using the Student’s *t*-test. Spearman’s rank correlation analysis was used to explore the correlations between different variables. LASSO regression, logistic regression, and nomogram development were conducted using “glmnet”, “survival”, and “rms” packages, respectively. *P* values < 0.05 were considered significant.

## Results

### Landscape of DMRGs in GDM Pregnancies

**Figure 2:**
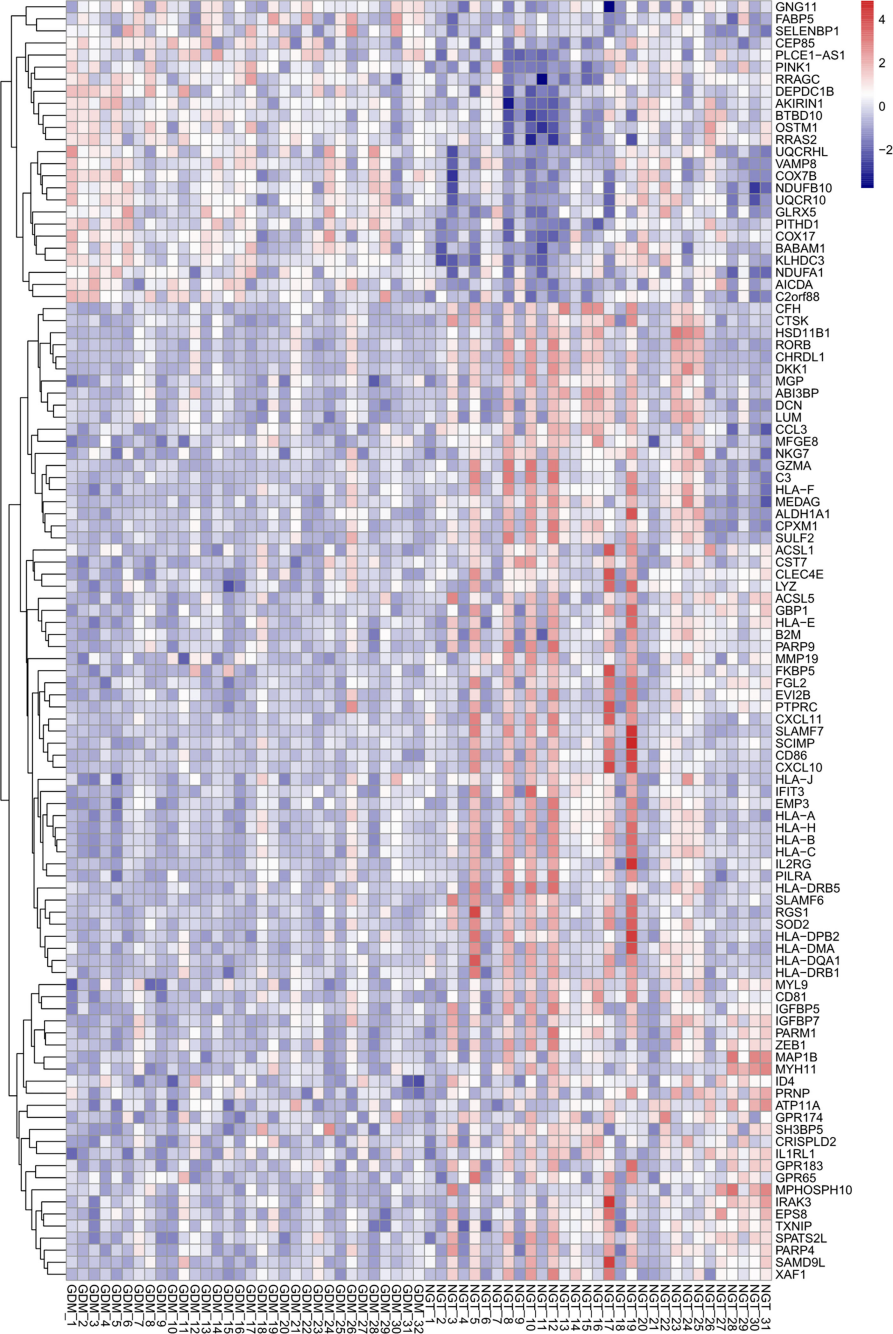
Heatmap of differentially expressed m^6^A-related genes. The up- and down-regulation of genes are indicated with red and blue color, respectively.

**Figure 3:**
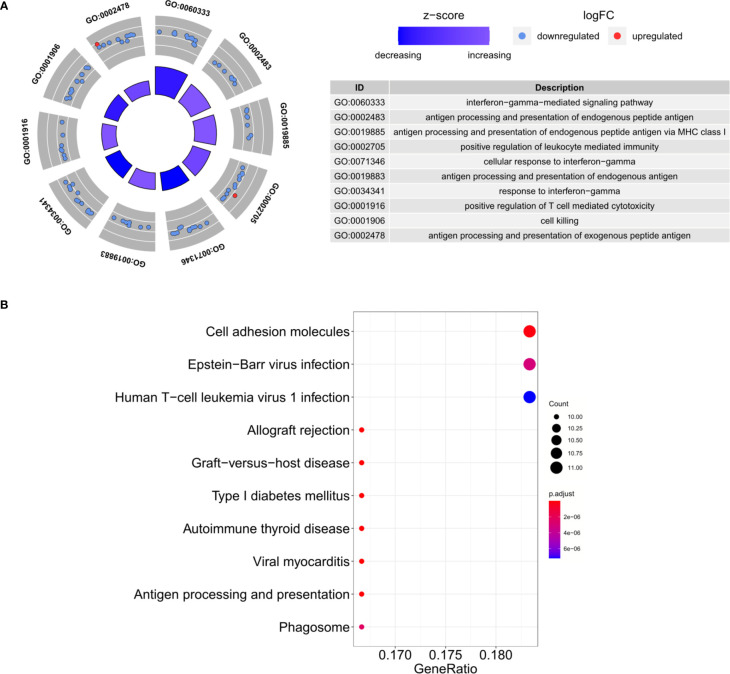
Functional enrichment analysis. **(A)** The top 10 GO biological process categories. **(B)** The top 10 KEGG pathways.

Based on the criteria described above, we identified 207 DEGs in GSE70493 and obtained 106 DMRGs by crossing the DEGs with 17,661 m^6^A-related genes. A heatmap of the DMRGs is shown in **Figure 2**. The list of all DMRGs is shown in **Table S1**. To elucidate the functions and pathways of the 106 DMRGs, we conducted enrichment analysis using R software. Based on the GO category biological process, DMRGs were mainly enriched for the terms interferon-gamma-mediated signaling pathway, antigen processing and presentation of endogenous antigen, positive regulation of leukocyte-mediated immunity, positive regulation of T cell-mediated cytotoxicity, cellular response to interferon-gamma, and cell killing (**Table S2** and **Figure 3A**). KEGG pathways were mainly enriched in viral myocarditis, type 1 diabetes mellitus (T1DM), phagosomes, cell adhesion molecules, and autoimmune thyroid disease (**Table S3** and **Figure 3B**).

### Immune Cell Infiltration in the Placenta

**Figure 4:**
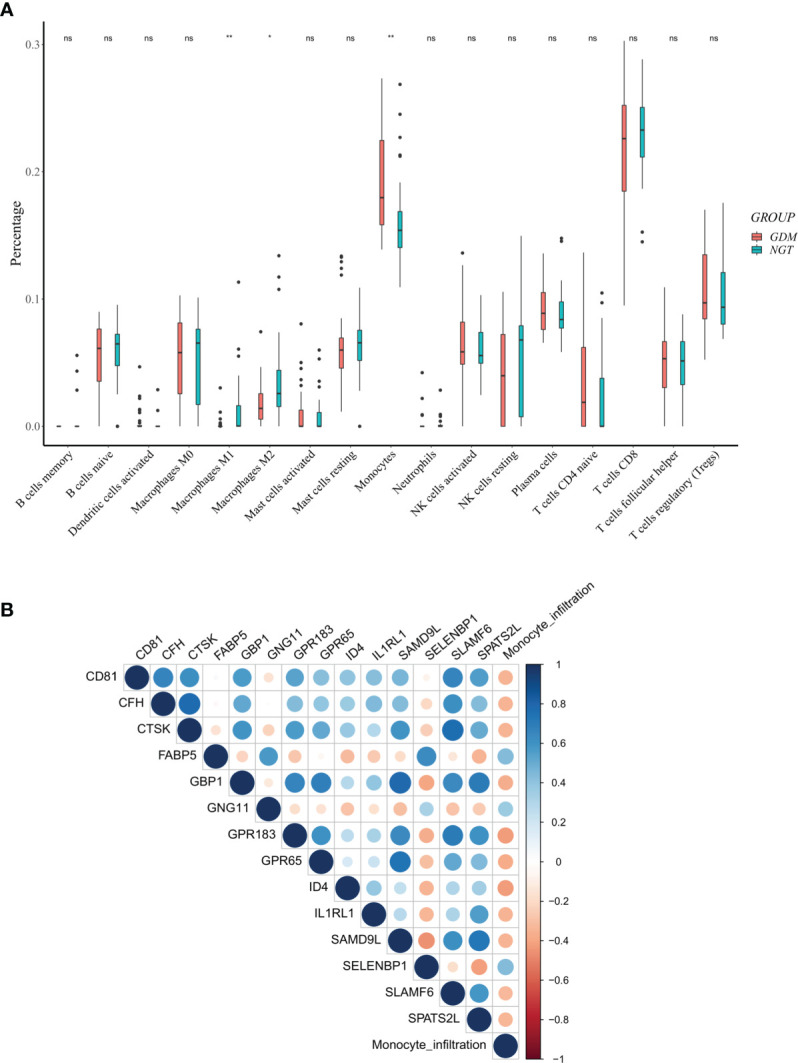
Differentially expressed m^6^A-related genes (DMRGs) related to monocyte infiltration in GDM Pregnancies. **(A)** Landscape of immune infiltrations in GDM Pregnancies. **(B)** Correlation among the abundance of monocytes and 14 correlated DMRGs. Asterisks denote statistical significance (ns, no significance; *p < 0.05; **p < 0.01).

We further explored differential immune cell infiltration in the placenta between GDM and control cases by quantifying the abundance of 22 immune cell types (**Supplementary File 1**). The results indicated that the infiltration of monocytes was higher in GDM placentas than in control samples, while the infiltration of macrophages M1 and M2 in GDM placentas were lower. No significant differences were observed among the other immune cells (**Figure 4A**). Higher propotion of M2 than M1 phenotype in GDM compared to controls was observed (**Supplementary Figure 1**).

### Identification of the DMRGs Signature Related to Monocyte Infiltration

**Figure 5:**
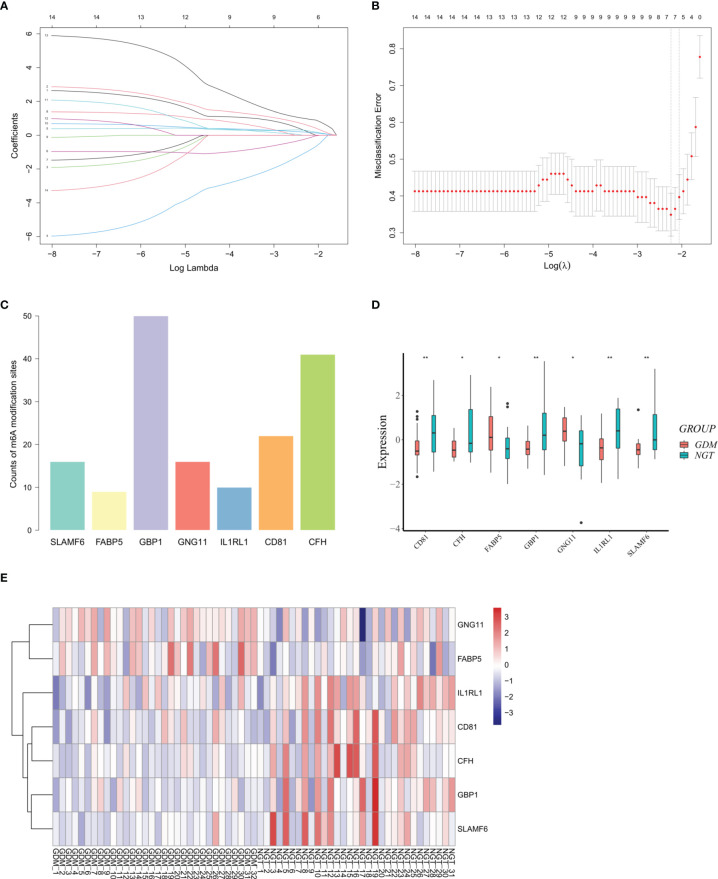
Differentially expressed m^6^A-related genes (DMRGs) signature selection through LASSO regression analysis. **(A)** LASSO coefficient profiles of 14 differentially expressed m^6^A-related genes (DMRGs). The coefficient profile plot was produced against the log (lambda). **(B)** The partial likelihood deviance (binomial deviance) curve was plotted versus log (lambda) to verify the optimal lambda value. Dotted vertical lines were drawn based on the 1-SE criteria. Seven DMRGs with non-zero coefficients were selected by optimal lambda. **(C)** Counts of potential m^6^A modification sites of the selected DMRGs. **(D)** Relative expression levels of *CD81, CFH, FABP5, GBP1, GNG11, IL1RL1*, and *SLAMF6*. **(E)** Hierarchical clustering of the expression pattern of *CD81, CFH, FABP5, GBP1, GNG11, IL1RL1*, and *SLAMF6*. Asterisks denote statistical significance ( *p < 0.05; **p < 0.01).

Considering the obviously high infiltration of monocytes in the GDM placentas, we calculated the PCCs of the abundance of monocytes and the expression levels of DMRGs. Fourteen DMRGs (methanethiol oxidase [*SELENBP1*], fatty acid-binding protein 5 [*FABP5*], G-protein coupled receptor 183 [*GPR183*], inhibitor of differentiation 4 [*ID4*], G-protein-coupled receptor 65 [*GPR65*], G-protein subunit γ 11 [*GNG11*], guanylate binding protein 1 [*GBP1*], complement factor H [*CFH*], tetraspanin [*CD81*], interleukin-1 receptor-like 1 [*IL1RL1*], cathepsin K [*CTSK*], sterile alpha motif domain-containing protein 9-like [*SAMD9L*], spermatogenesis associated serine-rich 2-like [*SPATS2L*], and signaling lymphocytic activation molecule family 6 [*SLAMF6*]), with a |PCC| > 0.3 and *P* value < 0.01, were selected from 106 DMRGs for further analysis (**Figure 4B**). Based on LASSO regression analysis, seven DMRGs (*CD81, CFH, FABP5, GBP1, GNG11, IL1RL1*, and *SLAMF6*) had nonzero coefficients, with a lambda coefficient of 0.1059 (**Figures 5A, B**). The count of the potential m^6^A modification sites are shown in **Figure 5C**. The expression matrix of the seven key DMRGs, based on the 63 samples, was extracted from the dataset GSE70493. The expression levels of *CD81, CFH, GBP1, IL1RL1*, and *SLAMF6* in GDM samples were lower than those in control samples, while expression levels of *FABP5* and *GNG11* were higher in GDM placentas than those in controls **(** **Figures 5D, E**). As shown in **Figure 4B**, these seven DMRGs were significantly correlated with each other.

### Development of the Nomogram Model

**Figure 6:**
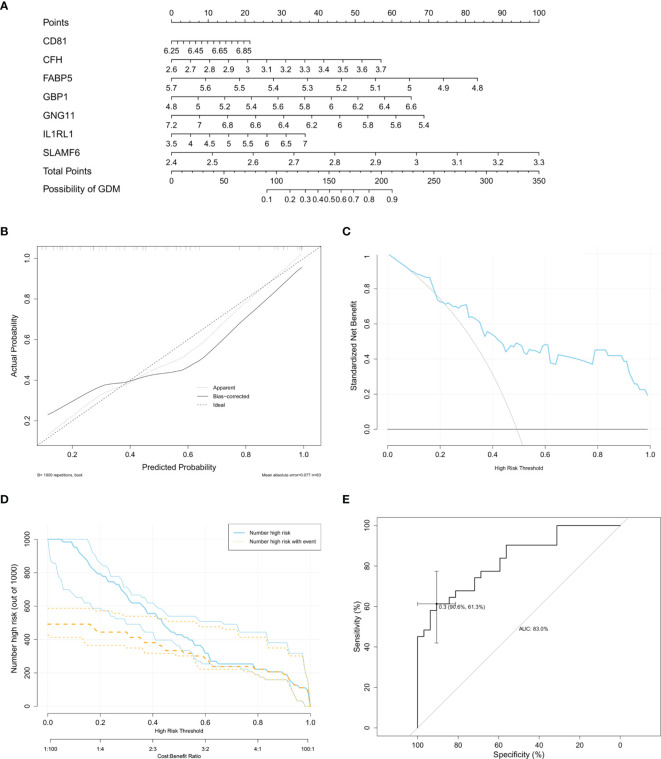
Development and internal validation of a nomogram model for GDM based on GSE70493. **(A)** Nomogram model for patients with GDM. **(B)** Calibration curve for predicting possibility of GDM. Decision curve **(C)** and clinical impact curve **(D)** for assessing the net benefit of the nomogram. **(E)** ROC curve to assess classifying ability of the nomogram model.

We extracted the expression matrix of the seven core DMRGs based on the training set of 63 samples extracted from dataset GSE70493. A model incorporating the DMRGs *CD81, CFH, FABP5, GBP1, GNG11, IL1RL1*, and *SLAMF6* was developed and presented as a nomogram (**Figure 6A**). The probability of GDM was accurately predicted using a calibration curve (**Figure 6B**). The decision curve (**Figure 6C**) and clinical impact curve (**Figure 6D**) revealed that our model demonstrated a positive net benefit without increasing the number of false positives. In addition, ROC curve analysis revealed that the area under the curve (AUC) was 83% (**Figure 6E**), indicating a good classification ability of the nomogram model.

### Diagnostic Value of the DMRGs Signature Related to Monocyte Infiltration

**Figure 7:**
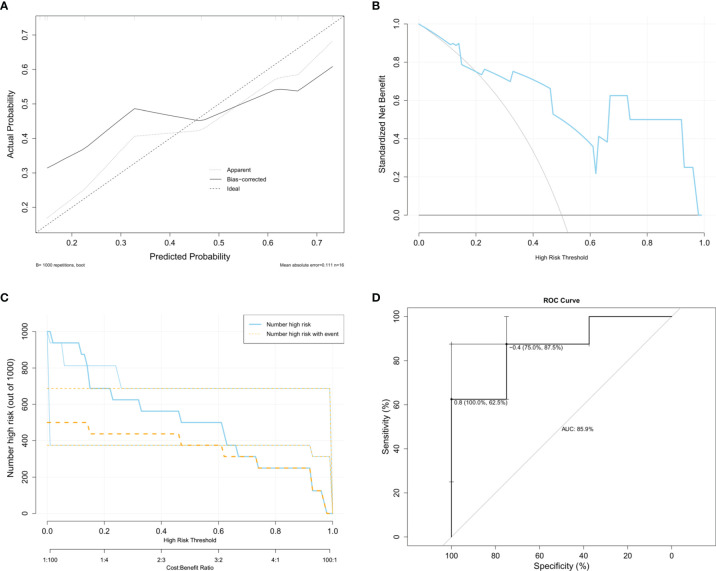
Diagnostic value of the monocyte infiltration related DMRGs signature based on GSE92772. **(A)** Calibration curve to identify the diagnostic value for GDM. Decision curve **(B)** and clinical impact curve **(C)** for assessing the clinical usage. **(D)** ROC curve used for assessing the sensitivity and specificity of the model.

Considering that screening for GDM is usually performed during 24–28 weeks of gestation, we selected GSE92772 as the validation set to evaluate the diagnostic value of the core DMRG signature, which is based on blood samples extracted during the second trimester. GSE92772 contains the expression matrix of *SLAMF6, FABP5, GBP1, GNG11, IL1RL1*, and *CD81*, without *CFH* present. In the validation set, the calibration curve (**Figure 7A**), decision curve **(** **Figure 7B**), and clinical impact curve (**Figure 7C**) also exhibited good performance. Moreover, the nomogram model exhibited high diagnostic value in distinguishing patients with GDM from those with NGT, with an AUC value of 85.9% (**Figure 7D**).

## Discussion

GDM is a common complication of pregnancy, adversely affecting both the mother and fetus (1, 2). The etiology of GDM, which involves genetic background and epigenetic modifications, remains unclear. Chronic low-grade inflammation during pregnancy can contribute to the pathogenesis of GDM by exacerbating maternal IR and inducing β cell failure (4). As an endocrine organ, the placenta derives inflammatory cytokines that stimulate immune cells and aggravates the immune/inflammatory response (12, 13). Moreover, the disturbance of immune cell infiltration in the placenta is attributed to pregnancy-specific diseases, including GDM, as well as GDM-related adverse outcomes (13–15). In this study, we found that the infiltration of monocytes was higher in GDM placentas than in control samples, while the infiltration level of macrophages (M1 and M2) in GDM placentas was lower than that in the controls. Monocyte infiltration has been shown to be crucial during inflammation. As important mediators of the innate immunity, monocytes circulate in the bloodstream and pass into tissues during the steady state and in increased quantities during inflammation (27). GDM is considered as a low-degree inflammation, and elevated levels of monocytes in the peripheral blood of patients with GDM have been previously reported (28). Based on the expression of superficial CD14 and CD16 in flow cytometry, monocytes can be divided into three subsets: classical (CD14++CD16-), intermediate (CD14+CD16++) and non-classical (CD14+CD16+) (29). Angelo et al. (28) observed increased percentage of classical monocytes, decreased frequency of intermediate monocytes in the peripheral blood of patients with GDM compared to controls. By contraries, an increase in the intermediate subset and a decreased frequency of classical monocytes were detected in healthy pregnancy compared to non-pregnant women (30). Considering that the variation in levels of monocyte subsets may contribute to the development of inflammation in GDM, it is essential to develop new studies on this topic to validate these findings. During gestation, bone marrow-derived monocytes can migrate from the bloodstream to the uterus and differentiate into decidua-specific macrophages upon exposure to this local microenvironment (31–34). A proportion of tissue-resident macrophages is constantly replaced by blood monocytes, and the mechanisms behind these differential renewal patterns are not fully understood and may be controlled by the tissue specific microenvironment (27, 35, 36). Inflammatory stimuli often depleted macrophages and induce monocyte recruitment; these monocytes might potentially contribute to tissue-resident macrophages upon the resolution of inflammation (27). Therefore, the decrease in macrophages and increased monocytes may be due to the inflammation during GDM. Decidual macrophages are highly plastic (37). It is generally accepted that macrophages are mainly the M1 (pro-inflammatory) phenotype during the pre-implantation period, and change to M2 (anti-inflammatory) phenotype following trophoblast attachment and invasion; macrophages seem to revert to M1 phenotype at the time of delivery (37–39). Inappropriate macrophage polarization may cause adverse pregnancy outcomes (30, 37). There are controversies regarding the use of placental macrophages in describing GDM. An imbalance of M2 to M1 macrophages has been observed in the placentas of diabetic patients and rats (40), as well as in placentas of GDM patients (41). Opposing conclusions have been reported in other studies, in which macrophages maintain the M2 phenotype in GDM compared to controls (42–44). In the present study, we also observed higher propotion of M2 than M1 phenotype in GDM compared to controls (**Supplementary Figure 1**).

m^6^A methylation plays a vital role in glucose/lipid metabolism as well as its related chronic inflammatory processes (23, 45–47). FTO moduates glucose metabolism *via* regulating forkhead box protein O1 and activating transcription factor 4 of m6A modification (46, 48). FTO also regulates adipogenesis by controlling cell cycle progression in an YTHDF2 dependent mechanism (45). METTL3 regulates lipid metabolism *via* mediating JAK1 mRNA stability an m6A-YTHDF2 dependent manner (47), and regulating NF-κB and MAPK *via* meditating m6A modification of TNF receptor associated factor 6 (49). Due to the dynamic and reversible nature, m6A methylation can be reversed by environmental stressors, including changes in nutrition. High-fat diet affecs METTL3 and FTO mRNA expression, and fasting state leads to the reduced FTO mRNA expression and increases m6A levels (50, 51). It remains unknown whether m^6^A modifications play a role in GDM. Exploration of the crosstalk between m6A modification and GDM may provide a potential strategy for the diagnosis, prognosis and treatment. We obtained m^6^A-related genes from the RMBase and RMvar databases and identified DMRGs based on the GSE70493 dataset. Enrichment analysis was conducted to determine the biological functions of the DMRGs. Notably, several pathways, such as type 1 diabetes mellitus and autoimmune thyroid disease, were closely correlated with the development and mal outcome of GDM. Recent studies have revealed that a small but significant population of patients with GDM develop postpartum T1DM (52, 53). Emerging evidence suggests that perturbations of the thyroid hormone signaling pathway and antibodies are associated with GDM development and adverse outcomes (54, 55). In terms of the GO biological process category, the DMRGs were closely related to inflammatory- and immune-related biological processes. Therefore, we suggest that, in addition to chronic inflammation, the immune response may also contribute to the pathophysiology of GDM.

As stated above, monocyte infiltration is aberrant in the placentas of patients with GDM. We obtained DMRGs related to monocyte infiltration, of which seven DMRGs (*CD81, CFH, FABP5, GBP1, GNG11, IL1RL1*, and *SLAMF6*) were selected through LASSO regression analysis to construct a nomogram. FABP5 belongs to the calycin superfamily and fatty-acid binding protein family, and serves as a gatekeeper for mitochondrial integrity to modulate regulatory T cells (Treg) and subdue immune responses (56). It has been reported that increased intra-tumoral FABP5 contributes to CD8+ T-cell infiltration and is linked to overall and recurrence-free survival, indicating that FABP5 could be an immunometabolic marker in hepatocellular carcinoma (57). Moreover, FABP5 has been observed to be enriched in classical monocytes of heart failure patients, suggesting that FABP5 contributes to monocyte activation (58). CD81 is a tetraspanin that participates in adaptive immunity and host-virus interactions (59, 60). As an inhibitor of the alternative complement pathway, CFH protects self-surfaces from immune attacks, thereby engaging in host-virus interactions and innate immunity (61–63). GBP1 is involved in macrophage apoptosis and pyroptosis (64). Interleukin-33 (IL-33) is the only known ligand of IL1RL1, and IL1RL1/IL-33 signaling participates in various inflammatory diseases (65). SLAMF6 is expressed in a variety of immune cells and may be involved in crosstalk between different microenvironments (66–68). Finally, GNG11, a member of the guanine nucleotide-binding protein family, is involved in various transmembrane signaling systems (69, 70). The nomogram showed a robust performance in distinguishing GDM patients from normal controls in the training set (GSE70493), with an AUC of 83%. GDM diagnosis is usually confirmed by a 75 g-oral glucose tolerance test during the second trimester. GSE92772, which is based on blood samples extracted during 24–28 weeks of gestation, was selected to externally validate the diagnostic capacity of the nomogram. The nomogram model exhibited a high diagnostic value with an AUC value of 85.9%, although it lacked the expression matrix of CFH. Therefore, our findings suggest that this m^6^A-related signature, correlated with monocyte infiltration, can be regarded as a novel biomarker and potential therapeutic target for GDM.

This study had a few limitations. A comprehensive analysis of the placenta and peripheral blood is warranted to verify the mRNA expression, protein expression and m^6^A-modification status of CD81, CFH, FABP5, GBP1, GNG11, IL1RL1, and SLAMF6. The diagnostic ability of the nomogram model may require further validation using a larger sample size. For subsequent research, more clinical parameters regarding valuable prognosis risk characteristics should be incorporated to verify the predictive ability of the nomogram.

## Conclusion

In this study, we analyzed the immune landscape and DMRGs in the placentas of patients with GDM. Some DMRGs were strongly associated with monocyte infiltration, which was higher in GDM placentas than in the control group. Based on seven selected DMRGs linked to monocyte infiltration in GDM placentas, we developed and validated a highly accurate nomogram for recognizing GDM.

## Data Availability Statement

The raw data of GSE70493 and GSE92772 were obtained from public databases (https://www.ncbi.nlm.nih.gov/geo/). The processed data are available from the corresponding author upon reasonable request.

## Author Contributions

RD: Conceptualization, Data curation, Formal analysis, Software, Writing—original draft. YW Conceptualization, Supervision, Validation, Writing—review and editing. LL: Funding acquisition, Methodology. All authors contributed to the article and approved the submitted version.

## Funding

The study was supported by the Clinical Research Project of Liaoning Diabetes Medical Nutrition Prevention Society [grant number LNSTNBYXYYFZXH-RS01A].

## Conflict of Interest

The authors declare that the research was conducted in the absence of any commercial or financial relationships that could be construed as a potential conflict of interest.

## Publisher’s Note

All claims expressed in this article are solely those of the authors and do not necessarily represent those of their affiliated organizations, or those of the publisher, the editors and the reviewers. Any product that may be evaluated in this article, or claim that may be made by its manufacturer, is not guaranteed or endorsed by the publisher.

## Supplementary Material

Supplementary Figure 1 Higher proportion of M2 than M1 phenotype of macrophages in GDM compared to controls.
