# Supplementary figures and images for: N6-Methyladenosine-Related Gene Signature Associated With Monocyte Infiltration Is Clinically Significant in Gestational Diabetes Mellitus
Source: Front Endocrinol (Lausanne). 2022 Mar 18;13:853857. doi: 10.3389/fendo.2022.853857 (PMC8971567; doi:10.3389/fendo.2022.853857)

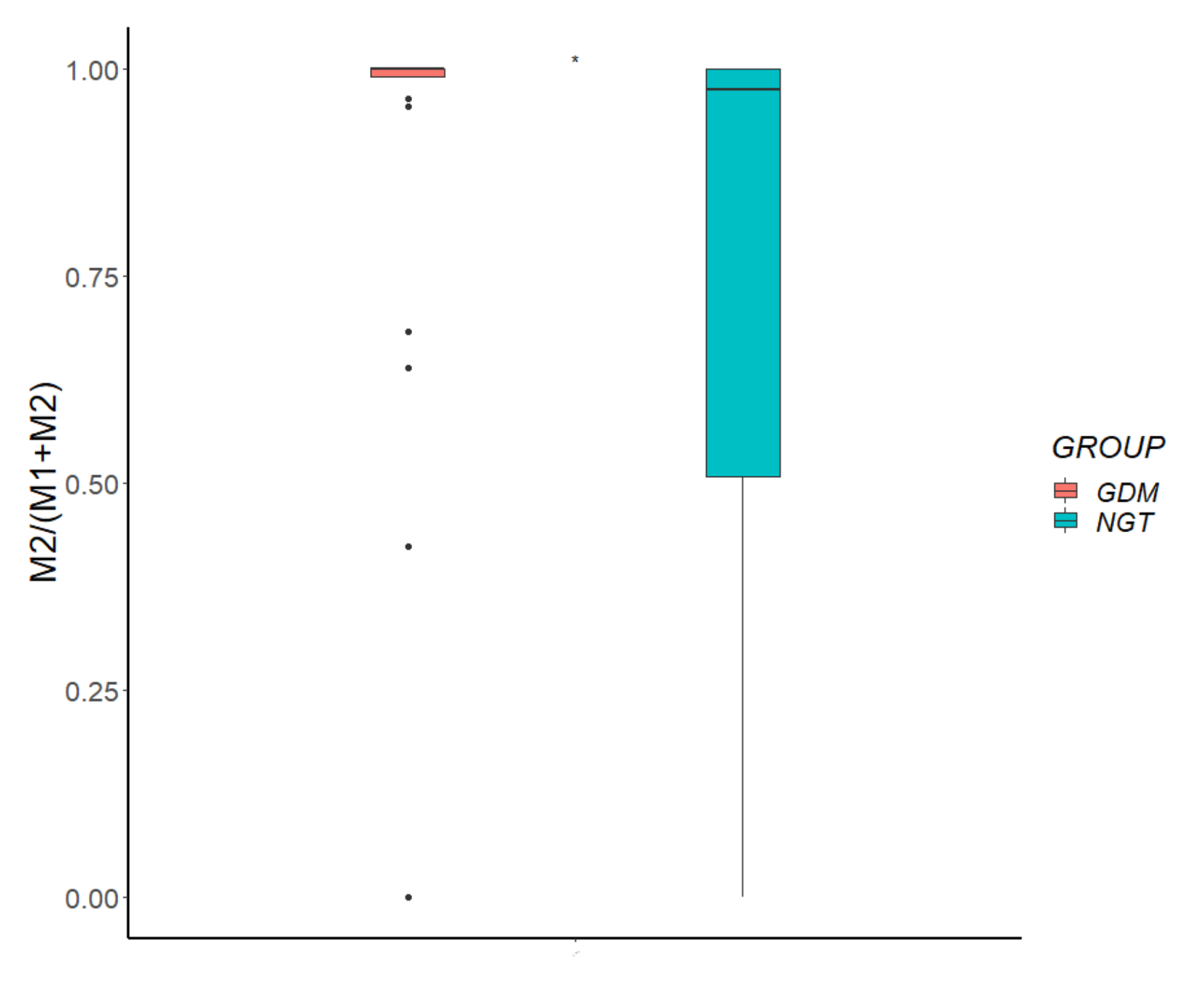

Supplement: Supplementary Figure 1 — Higher proportion of M2 than M1 phenotype of macrophages in GDM compared to controls. [file Image_1.tif]
