# Supplementary material for: N6-Methyladenosine-Related Gene Signature Associated With Monocyte Infiltration Is Clinically Significant in Gestational Diabetes Mellitus
Source: Front Endocrinol (Lausanne). 2022 Mar 18;13:853857. doi: 10.3389/fendo.2022.853857 (PMC8971567; doi:10.3389/fendo.2022.853857)
Supplement: Supplementary file 2 [file DataSheet_1.pdf]

CIBERSORTx Output Display

Input Sample	B cells naive	B cells memory	Plasma cells	T cells CD8	T cells CD4 naive	T cells CD4 memory resting	T cells CD4 memory activated	T cells follicular helper	T cells regulatory (Tregs)	T cells gamma delta	NK cells resting	NK cells activated	Monocytes	Macrophages M0	Macrophages M1	Macrophages M2	Dendritic cells resting	Dendritic cells activated	Mast cells resting	Mast cells activated	Eosinophils	Neutrophils	P-value	Correlation	RMSE
GSM1784987	0.078	0	0.074	0.262	0	0	0	0.081	0.082	0	0.063	0.049	0.157	0.09	0	0.009	0	0	0.055	0	0	0	0.790	-0.011	1.100
GSM1784988	0.061	0	0.077	0.303	0	0	0	0.072	0.085	0	0.072	0.046	0.152	0.086	0	0	0	0	0.046	0	0	0	0.780	-0.011	1.107
GSM1784989	0.069	0	0.081	0.299	0	0	0	0.061	0.096	0	0.084	0.042	0.139	0.07	0	0.025	0	0	0.036	0	0	0	0.760	-0.010	1.107
GSM1784990	0.025	0	0.092	0.24	0.077	0	0	0	0.156	0	0.015	0.055	0.192	0.015	0.023	0.021	0	0	0.052	0.035	0	0	0.700	-0.006	1.095
GSM1784991	0.064	0	0.129	0.123	0.071	0	0	0.032	0.098	0	0	0.096	0.197	0.047	0	0.016	0	0.003	0.124	0	0	0	0.640	-0.002	1.088
GSM1784993	0	0.043	0.076	0.212	0.025	0	0	0.034	0.154	0	0	0.065	0.269	0.017	0	0.02	0	0	0.032	0.053	0	0	0.650	-0.003	1.092
GSM1784994	0.061	0	0.077	0.231	0	0	0	0.087	0.076	0	0.098	0.037	0.159	0.084	0	0.016	0	0	0.073	0	0	0	0.760	-0.010	1.096
GSM1784995	0.052	0	0.08	0.269	0	0	0	0.065	0.085	0	0.105	0.025	0.187	0.089	0	0	0	0	0.043	0	0	0	0.790	-0.012	1.102
GSM1784996	0.013	0	0.103	0.186	0.06	0	0	0.013	0.149	0	0.004	0.083	0.273	0.013	0.002	0.043	0	0	0.059	0	0	0	0.730	-0.008	1.094
GSM1784997	0.063	0	0.066	0.249	0	0	0	0.058	0.115	0	0.059	0.064	0.148	0.103	0.001	0	0	0	0.074	0	0	0	0.730	-0.008	1.097
GSM1784998	0.072	0	0.066	0.288	0	0	0	0.054	0.102	0	0.085	0.051	0.141	0.058	0.002	0.019	0	0	0.061	0	0	0	0.780	-0.011	1.107
GSM1784999	0.027	0	0.114	0.207	0.1	0	0	0.009	0.122	0	0.031	0	0.238	0.028	0	0.018	0	0.013	0.012	0.081	0	0	0.650	-0.003	1.089
GSM1785000	0.037	0	0.073	0.237	0	0	0	0.086	0.08	0	0.077	0.057	0.168	0.079	0	0.04	0	0	0.065	0	0	0	0.730	-0.008	1.095
GSM1785001	0.043	0	0.079	0.261	0	0	0	0.081	0.09	0	0.077	0.059	0.163	0.091	0	0.001	0	0	0.055	0	0	0	0.690	-0.005	1.101
GSM1785003	0	0.028	0.145	0.145	0	0	0	0.038	0.116	0	0.013	0.077	0.154	0.065	0.113	0.014	0.006	0	0.073	0.011	0	0	0.390	0.026	1.080
GSM1785004	0.052	0	0.079	0.237	0	0	0	0.084	0.078	0	0.079	0.051	0.168	0.101	0	0.005	0	0	0.066	0	0	0	0.710	-0.007	1.095
GSM1785005	0.057	0	0.084	0.262	0	0	0	0.079	0.083	0	0.076	0.044	0.149	0.089	0	0.022	0	0	0.055	0	0	0	0.730	-0.008	1.098
GSM1785006	0.078	0	0.091	0.251	0	0	0	0.057	0.072	0	0.069	0.051	0.138	0.065	0.04	0.031	0	0	0.058	0	0	0	0.370	0.030	1.080
GSM1785007	0.044	0	0.072	0.227	0	0	0	0.059	0.071	0	0.053	0.06	0.179	0.059	0	0.047	0	0	0.129	0	0	0	0.790	-0.012	1.089
GSM1785008	0.061	0	0.068	0.201	0.013	0	0	0.048	0.134	0	0.026	0.077	0.244	0.018	0.001	0.028	0	0	0.068	0.012	0	0	0.690	-0.005	1.093
GSM1785009	0.09	0	0.136	0.189	0.031	0	0	0.027	0.112	0	0	0.083	0.232	0.025	0	0.01	0	0	0.065	0	0	0	0.680	-0.005	1.100
GSM1785011	0	0	0.101	0.214	0.13	0	0	0.027	0.096	0	0.01	0.054	0.228	0.026	0.011	0.02	0	0	0.047	0.037	0	0	0.700	-0.006	1.093
GSM1785012	0.057	0	0.078	0.253	0	0	0	0.065	0.091	0	0.072	0.061	0.148	0.081	0	0.032	0	0	0.062	0	0	0	0.800	-0.013	1.100
GSM1785013	0.048	0	0.1	0.225	0.058	0	0	0	0.099	0	0.068	0.029	0.245	0.05	0	0	0	0.029	0.028	0.02	0	0	0.630	-0.001	1.091
GSM1785014	0.066	0	0.102	0.25	0	0	0	0.046	0.073	0	0.078	0.056	0.14	0.067	0.037	0.03	0	0	0.055	0	0	0	0.350	0.034	1.081
GSM1785015	0.079	0	0.071	0.249	0	0	0	0.093	0.055	0	0.062	0.049	0.166	0.082	0	0.031	0	0	0.063	0	0	0	0.750	-0.009	1.094
GSM1785016	0.063	0	0.084	0.27	0	0	0	0.065	0.086	0	0.069	0.065	0.142	0.078	0	0.025	0	0	0.055	0	0	0	0.810	-0.014	1.105
GSM1785017	0.067	0	0.08	0.256	0	0	0	0.088	0.083	0	0.06	0.061	0.168	0.099	0	0	0	0	0.038	0	0	0	0.590	0.001	1.097
GSM1785018	0.078	0	0.095	0.173	0.03	0	0	0.037	0.118	0	0.013	0.077	0.23	0.064	0	0.001	0	0.024	0.058	0	0	0	0.470	0.014	1.082
GSM1785020	0.07	0	0.109	0.242	0	0	0	0.039	0.069	0	0.079	0.054	0.143	0.065	0.036	0.043	0	0	0.05	0	0	0	0.350	0.035	1.080
GSM1785021	0.047	0	0.086	0.214	0.097	0	0	0.043	0.119	0	0	0.075	0.135	0.007	0	0.051	0	0	0.087	0.031	0	0.008	0.720	-0.008	1.095
GSM1785022	0.043	0	0.072	0.211	0	0	0	0.066	0.124	0	0.102	0.048	0.154	0.081	0.017	0	0	0	0.081	0	0	0	0.700	-0.006	1.095
GSM1785023	0.066	0	0.13	0.135	0.11	0	0	0.015	0.089	0	0	0.082	0.195	0.056	0	0.003	0	0	0.119	0	0	0	0.630	-0.001	1.088
GSM1785024	0.096	0	0.098	0.194	0.105	0	0	0.028	0.071	0	0	0.082	0.126	0	0.003	0.117	0	0	0.072	0.001	0	0.008	0.660	-0.004	1.091
GSM1785025	0.09	0	0.085	0.219	0.043	0	0	0	0.092	0	0	0.088	0.13	0	0	0.134	0	0	0.109	0	0	0.009	0.700	-0.006	1.088
GSM1785027	0.072	0	0.072	0.199	0	0	0	0.031	0.094	0	0.065	0.037	0.154	0.069	0.055	0.057	0	0	0.096	0	0	0	0.540	0.007	1.076
GSM1785028	0.073	0	0.078	0.262	0	0	0	0.067	0.069	0	0.063	0.052	0.15	0.092	0	0.017	0	0	0.076	0	0	0	0.800	-0.012	1.097
GSM1785029	0.069	0	0.07	0.232	0	0	0	0.051	0.085	0	0.068	0.041	0.122	0.043	0.061	0.107	0	0	0.049	0	0	0	0.540	0.006	1.083
GSM1785030	0.085	0	0.082	0.251	0	0	0	0.109	0.053	0	0.084	0.049	0.156	0.092	0	0.016	0	0	0.023	0	0	0	0.690	-0.005	1.103
GSM1785031	0.056	0	0.084	0.256	0	0	0	0.07	0.078	0	0.061	0.073	0.16	0.07	0	0.047	0	0	0.044	0	0	0	0.810	-0.014	1.102
GSM1785032	0.02	0	0.123	0.133	0.054	0	0	0.054	0.17	0	0	0.127	0.179	0.027	0	0	0	0.022	0.085	0.005	0	0	0.780	-0.011	1.100
GSM1785033	0.065	0	0.115	0.153	0.067	0	0	0.082	0.133	0	0	0.074	0.227	0	0	0.025	0	0	0	0.06	0	0	0.750	-0.009	1.101
GSM1785035	0.051	0	0.112	0.188	0.085	0	0	0.045	0.104	0	0	0.103	0.169	0.017	0.015	0.026	0	0	0.08	0	0	0.004	0.700	-0.006	1.096
GSM1785036	0	0	0.081	0.224	0.032	0	0	0.04	0.163	0	0	0.095	0.212	0	0	0.074	0	0	0.06	0.019	0	0	0.700	-0.006	1.096
GSM1785037	0	0.056	0.058	0.238	0	0	0	0.022	0.176	0	0.08	0.069	0.109	0.071	0.006	0	0	0.012	0.072	0.021	0	0.009	0.510	0.009	1.096
GSM1785038	0.066	0	0.098	0.261	0.07	0	0	0.001	0.113	0	0.031	0.048	0.18	0.017	0	0.033	0	0	0.071	0.011	0	0	0.640	-0.002	1.096
GSM1785039	0.076	0	0.112	0.197	0.028	0	0	0.048	0.136	0	0	0.087	0.215	0.026	0.003	0.006	0	0	0.051	0.015	0	0	0.720	-0.007	1.099
GSM1785040	0.056	0	0.068	0.258	0	0	0	0.088	0.088	0	0.072	0.06	0.152	0.072	0	0.043	0	0	0.043	0	0	0	0.830	-0.015	1.103
GSM1785041	0.016	0	0.116	0.199	0.042	0	0	0.056	0.143	0	0.045	0.048	0.18	0.007	0	0.005	0	0.047	0.026	0.045	0	0.022	0.650	-0.003	1.093
GSM1785042	0.063	0	0.066	0.179	0	0	0	0.033	0.142	0	0.106	0.045	0.224	0.07	0.006	0	0	0	0.064	0.003	0	0	0.710	-0.007	1.094
GSM1785044	0.077	0	0.077	0.236	0	0	0	0.081	0.075	0	0.07	0.058	0.146	0.087	0	0.023	0	0	0.069	0	0	0	0.810	-0.014	1.098
GSM1785045	0.025	0	0.103	0.252	0.037	0	0	0.022	0.149	0	0	0.056	0.17	0	0	0.074	0	0.004	0.016	0.05	0	0.042	0.550	0.005	1.090
GSM1785046	0.085	0	0.12	0.099	0.08	0	0	0	0.117	0	0	0.092	0.187	0.025	0.03	0.022	0	0	0.133	0	0	0.009	0.590	0.001	1.085
GSM1785047	0.043	0	0.082	0.278	0	0	0	0.081	0.077	0	0.074	0.053	0.179	0.093	0	0.007	0	0	0.034	0	0	0	0.720	-0.007	1.103
GSM1785048	0	0	0.101	0.095	0.068	0	0	0.011	0.153	0	0.024	0.136	0.191	0.057	0	0.009	0	0.013	0.134	0	0	0.009	0.530	0.008	1.086
GSM1785049	0.063	0	0.083	0.214	0	0	0	0.061	0.15	0	0.089	0.056	0.122	0.077	0.005	0	0	0	0.079	0	0	0.001	0.750	-0.009	1.101
GSM1785050	0.07	0	0.078	0.233	0	0	0	0.064	0.082	0	0.093	0.056	0.142	0.075	0	0.037	0	0	0.07	0	0	0	0.800	-0.013	1.099
GSM1785052	0.078	0	0.101	0.166	0.137	0	0	0.037	0.052	0	0.034	0.008	0.244	0.051	0	0.01	0	0	0.05	0.032	0	0	0.580	0.003	1.086
GSM1785053	0.072	0	0.148	0.198	0	0	0	0	0.122	0	0.002	0.087	0.213	0.018	0.002	0.045	0	0	0.088	0.002	0	0.002	0.610	0.001	1.095
GSM1785054	0.075	0	0.076	0.239	0	0	0	0.056	0.086	0	0.103	0.046	0.157	0.068	0	0.043	0	0	0.051	0	0	0	0.800	-0.013	1.101
GSM1785055	0.079	0	0.094	0.225	0.089	0	0	0.052	0.102	0	0	0.053	0.173	0.022	0	0.009	0	0.011	0.065	0.027	0	0	0.720	-0.007	1.096
GSM1785056	0.051	0	0.091	0.187	0	0	0	0.062	0.097	0	0.15	0.025	0.149	0.04	0.014	0.032	0	0	0.075	0	0	0.028	0.810	-0.014	1.099
GSM1785057	0.032	0	0.094	0.233	0.025	0	0	0.036	0.147	0	0	0.085	0.239	0.014	0	0.013	0	0	0.061	0.023	0	0	0.780	-0.011	1.100
