# Supplementary material for: N6-Methyladenosine-Related Gene Signature Associated With Monocyte Infiltration Is Clinically Significant in Gestational Diabetes Mellitus
Source: Front Endocrinol (Lausanne). 2022 Mar 18;13:853857. doi: 10.3389/fendo.2022.853857 (PMC8971567; doi:10.3389/fendo.2022.853857)
Supplement: Supplementary file 3 [file Table_1.docx]

**Table S1.** The list of differentially expressed m6A-related genes in GSE70493.

| Gene | *P* Value | logFC | Probe ID |
| --- | --- | --- | --- |
| *ABI3BP* | 0.0017501 | -0.436 | TC06000328.hg.1 |
| *ACSL1* | 0.0093121 | -0.107 | TC6_qbl_hap6000146.hg.1 |
| *ACSL5* | 0.00216 | -0.182 | TC07001606.hg.1 |
| *AICDA* | 0.0038231 | 0.144 | TC6_mann_hap4000188.hg.1 |
| *AKIRIN1* | 0.0041794 | 0.124 | TC01003403.hg.1 |
| *ALDH1A1* | 0.0061529 | -0.205 | TC6_mcf_hap5000023.hg.1 |
| *ATP11A* | 0.0079253 | -0.202 | TC0X001121.hg.1 |
| *B2M* | 0.0095968 | -0.242 | TC20000164.hg.1 |
| *BABAM1* | 0.0021646 | 0.135 | TC13000103.hg.1 |
| *BTBD10* | 0.0096317 | 0.147 | TC0X000428.hg.1 |
| *C2orf88* | 0.0008056 | 0.149 | TC03000626.hg.1 |
| *C3* | 0.009684 | -0.216 | TC12001282.hg.1 |
| *CCL3* | 0.0069195 | -0.124 | TC01001622.hg.1 |
| *CD81* | 0.0022249 | -0.109 | TC19001776.hg.1 |
| *CD86* | 0.0028208 | -0.117 | TC6_ssto_hap7000016.hg.1 |
| *CEP85* | 0.0012236 | 0.195 | TC04001226.hg.1 |
| *CFH* | 0.0010811 | -0.213 | TC6_dbb_hap3000210.hg.1 |
| *CHRDL1* | 0.001317 | -0.34 | TC16000660.hg.1 |
| *CLEC4E* | 0.000581 | -0.148 | TC01000339.hg.1 |
| *COX17* | 0.002023 | 0.145 | TC6_ssto_hap7000086.hg.1 |
| *COX7B* | 0.0011653 | 0.15 | TC06004060.hg.1 |
| *CPXM1* | 0.0067775 | -0.18 | TC01003211.hg.1 |
| *CRISPLD2* | 0.0058182 | -0.187 | TC06004059.hg.1 |
| *CST7* | 0.0037432 | -0.152 | TC13000491.hg.1 |
| *CTSK* | 0.0025786 | -0.221 | TC01001090.hg.1 |
| *CXCL10* | 0.0033137 | -0.715 | TC6_ssto_hap7000029.hg.1 |
| *CXCL11* | 0.0051727 | -0.163 | TC6_qbl_hap6000015.hg.1 |
| *DCN* | 0.0095637 | -0.357 | TC01000262.hg.1 |
| *DEPDC1B* | 0.002502 | 0.315 | TC06004122.hg.1 |
| *DKK1* | 0.0036626 | -0.65 | TC6_ssto_hap7000020.hg.1 |
| *EMP3* | 0.0060402 | -0.276 | TC08000518.hg.1 |
| *EPS8* | 0.0060426 | -0.135 | TC6_ssto_hap7000190.hg.1 |
| *EVI2B* | 0.0063217 | -0.198 | TC20000913.hg.1 |
| *FABP5* | 0.0045821 | 0.125 | TC03003390.hg.1 |
| *FGL2* | 0.0002291 | -0.398 | TC14000523.hg.1 |
| *FKBP5* | 0.0006788 | -0.133 | TC05000336.hg.1 |
| *GBP1* | 0.0011636 | -0.249 | TC6_cox_hap2000020.hg.1 |
| *GLRX5* | 0.0013707 | 0.105 | TC19000699.hg.1 |
| *GNG11* | 0.0072313 | 0.221 | TC0X001278.hg.1 |
| *GPR174* | 0.0015139 | -0.128 | TC09001211.hg.1 |
| *GPR183* | 0.0057244 | -0.168 | TC07001546.hg.1 |
| *GPR65* | 0.0092163 | -0.154 | TC05001392.hg.1 |
| *GZMA* | 0.0054412 | -0.191 | TC03001207.hg.1 |
| *HLA-A* | 0.0003393 | -0.183 | TC6_cox_hap2000237.hg.1 |
| *HLA-B* | 0.0003018 | -0.295 | TC6_apd_hap1000101.hg.1 |
| *HLA-C* | 0.0000097 | -0.263 | TC6_dbb_hap3000214.hg.1 |
| *HLA-DMA* | 0.0075301 | -0.295 | TC6_mann_hap4000017.hg.1 |
| *HLA-DPB2* | 0.0017905 | -0.121 | TC15000342.hg.1 |
| *HLA-DQA1* | 0.0005565 | -0.261 | TC06000338.hg.1 |
| *HLA-DRB1* | 0.0094932 | -0.557 | TC6_ssto_hap7000164.hg.1 |
| *HLA-DRB5* | 0.0091053 | -0.61 | TC06000400.hg.1 |
| *HLA-E* | 0.002126 | -0.23 | TC6_mcf_hap5000194.hg.1 |
| *HLA-F* | 0.0021409 | -0.131 | TC06000323.hg.1 |
| *HLA-H* | 0.0006769 | -0.224 | TC13000411.hg.1 |
| *HLA-J* | 0.0029668 | -0.18 | TC01001640.hg.1 |
| *HSD11B1* | 0.0034767 | -0.351 | TC12000611.hg.1 |
| *ID4* | 0.0093362 | -0.114 | TC01006291.hg.1 |
| *IFIT3* | 0.0066383 | -0.113 | TC04001809.hg.1 |
| *IGFBP5* | 0.0075289 | -0.222 | TC02002758.hg.1 |
| *IGFBP7* | 0.0019296 | -0.362 | TC06000409.hg.1 |
| *IL1RL1* | 0.0014916 | -0.605 | TC6_cox_hap2000091.hg.1 |
| *IL2RG* | 0.0037069 | -0.158 | TC6_mann_hap4000022.hg.1 |
| *IRAK3* | 0.0022072 | -0.136 | TC20000051.hg.1 |
| *KLHDC3* | 0.0023751 | 0.122 | TC6_dbb_hap3000029.hg.1 |
| *LUM* | 0.0067244 | -0.407 | TC10000219.hg.1 |
| *LYZ* | 0.0096077 | -0.427 | TC11001442.hg.1 |
| *MAP1B* | 0.003674 | -0.127 | TC02000428.hg.1 |
| *MEDAG* | 0.0078724 | -0.115 | TC6_apd_hap1000017.hg.1 |
| *MFGE8* | 0.001106 | -0.158 | TC6_ssto_hap7000191.hg.1 |
| *MGP* | 0.0052329 | -0.237 | TC10000350.hg.1 |
| *MMP19* | 0.0025648 | -0.131 | TC12001276.hg.1 |
| *MPHOSPH10* | 0.0091368 | -0.117 | TC19001103.hg.1 |
| *MYH11* | 0.0094568 | -0.137 | TC20000268.hg.1 |
| *MYL9* | 0.0073039 | -0.209 | TC0X000423.hg.1 |
| *NDUFA1* | 0.0074835 | 0.142 | TC07000556.hg.1 |
| *NDUFB10* | 0.0024489 | 0.146 | TC6_cox_hap2000236.hg.1 |
| *NKG7* | 0.0070897 | -0.104 | TC6_mann_hap4000031.hg.1 |
| *OSTM1* | 0.0087463 | 0.105 | TC6_apd_hap1000118.hg.1 |
| *PARM1* | 0.0010876 | -0.253 | TC09000338.hg.1 |
| *PARP4* | 0.0053855 | -0.183 | TC12001803.hg.1 |
| *PARP9* | 0.005157 | -0.144 | TC01000301.hg.1 |
| *PILRA* | 0.0042823 | -0.115 | TC12001804.hg.1 |
| *PINK1* | 0.0046325 | 0.108 | TC01000491.hg.1 |
| *PITHD1* | 0.0007041 | 0.128 | TC6_mcf_hap5000195.hg.1 |
| *PLCE1-AS1* | 0.0092473 | 0.375 | TC02000512.hg.1 |
| *PRNP* | 0.0047201 | -0.132 | TC02004994.hg.1 |
| *PTPRC* | 0.0063065 | -0.197 | TC6_cox_hap2000036.hg.1 |
| *RGS1* | 0.0029737 | -0.206 | TC04001305.hg.1 |
| *RORB* | 0.0053544 | -0.164 | TC01001750.hg.1 |
| *RRAGC* | 0.0066985 | 0.115 | TC01002251.hg.1 |
| *RRAS2* | 0.0063666 | 0.143 | TC22000202.hg.1 |
| *SAMD9L* | 0.0025411 | -0.288 | TC03001705.hg.1 |
| *SCIMP* | 0.0051695 | -0.109 | TC01006351.hg.1 |
| *SELENBP1* | 0.008687 | 0.138 | TC6_mcf_hap5000207.hg.1 |
| *SH3BP5* | 0.0051536 | -0.163 | TC6_mcf_hap5000206.hg.1 |
| *SLAMF6* | 0.0008443 | -0.142 | TC6_cox_hap2000023.hg.1 |
| *SLAMF7* | 0.003405 | -0.2 | TC11000063.hg.1 |
| *SOD2* | 0.0011125 | -0.211 | TC6_ssto_hap7000217.hg.1 |
| *SPATS2L* | 0.0031125 | -0.122 | TC06004141.hg.1 |
| *SULF2* | 0.0062931 | -0.122 | TC6_cox_hap2000026.hg.1 |
| *TXNIP* | 0.007294 | -0.266 | TC6_qbl_hap6000082.hg.1 |
| *UQCR10* | 0.0096853 | 0.134 | TC20000565.hg.1 |
| *UQCRHL* | 0.0025286 | 0.157 | TC01001372.hg.1 |
| *VAMP8* | 0.0045298 | 0.136 | TC12001178.hg.1 |
| *XAF1* | 0.0056583 | -0.156 | TC02001115.hg.1 |
| *ZEB1* | 0.006692 | -0.127 | TC11001434.hg.1 |

**Table S2.** The top 10 enriched GO biological process categories of the differentially expressed m6A-related genes in GDM.

| **ID** | **GO Term** | **Ajusted *P* Value** | **Z Score** | **Genes** | **Gene Count** |
| --- | --- | --- | --- | --- | --- |
| GO:0060333 | interferon-gamma-mediated signaling pathway | 1.13E-10 | -3.464101615 | *B2M,GBP1,HLA-A,HLA-B,HLA-C,HLA-DQA1,HLA-DRB1,HLA-DRB5,HLA-E,HLA-F,HLA-H,PARP9* | 12 |
| GO:0002483 | antigen processing and presentation of endogenous peptide antigen | 1.26E-09 | -2.645751311 | *B2M,HLA-A,HLA-B,HLA-C,HLA-E,HLA-F,HLA-H* | 7 |
| GO:0019885 | antigen processing and presentation of endogenous peptide antigen via MHC class I | 1.26E-09 | -2.645751311 | *B2M,HLA-A,HLA-B,HLA-C,HLA-E,HLA-F,HLA-H* | 7 |
| GO:0002705 | positive regulation of leukocyte mediated immunity | 4.47E-09 | -2.886751346 | *B2M,C3,CD81,HLA-A,HLA-B,HLA-E,HLA-F,HLA-H,PTPRC,SCIMP,SLAMF6,VAMP8* | 12 |
| GO:0071346 | cellular response to interferon-gamma | 5.64E-09 | -3.605551275 | *B2M,CCL3,GBP1,HLA-A,HLA-B,HLA-C,HLA-DQA1,HLA-DRB1,HLA-DRB5,HLA-E,HLA-F,HLA-H,PARP9* | 13 |
| GO:0019883 | antigen processing and presentation of endogenous antigen | 1.09E-08 | -2.645751311 | *B2M,HLA-A,HLA-B,HLA-C,HLA-E,HLA-F,HLA-H* | 7 |
| GO:0034341 | response to interferon-gamma | 1.50E-08 | -3.605551275 | *B2M,CCL3,GBP1,HLA-A,HLA-B,HLA-C,HLA-DQA1,HLA-DRB1,HLA-DRB5,HLA-E,HLA-F,HLA-H,PARP9* | 13 |
| GO:0001916 | positive regulation of T cell mediated cytotoxicity | 2.67E-08 | -2.645751311 | *B2M,HLA-A,HLA-B,HLA-E,HLA-F,HLA-H,PTPRC* | 7 |
| GO:0001906 | cell killing | 2.67E-08 | -3.464101615 | *B2M,C3,CFH,HLA-A,HLA-B,HLA-E,HLA-F,HLA-H,LYZ,PTPRC,SLAMF6,SLAMF7* | 12 |
| GO:0002478 | antigen processing and presentation of exogenous peptide antigen | 3.28E-08 | -2.886751346 | *B2M,HLA-A,HLA-B,HLA-C,HLA-DMA,HLA-DQA1,HLA-DRB1,HLA-DRB5,HLA-E,HLA-F,HLA-H,VAMP8* | 12 |

**Table S3.** The top 10 enriched KEGG pathways of the differentially expressed m6A-related genes in GDM.

| **ID** | **Description** | **Ajusted *P* Value** | **Genes** | **Gene Count** |
| --- | --- | --- | --- | --- |
| hsa05330 | Allograft rejection | 1.23E-11 | *CD86/HLA-A/HLA-B/HLA-C/HLA-DMA/HLA-DQA1/HLA-DRB1/HLA-DRB5/HLA-E/HLA-F* | 10 |
| hsa05332 | Graft-versus-host disease | 1.61E-11 | *CD86/HLA-A/HLA-B/HLA-C/HLA-DMA/HLA-DQA1/HLA-DRB1/HLA-DRB5/HLA-E/HLA-F* | 10 |
| hsa04940 | Type I diabetes mellitus | 1.61E-11 | *CD86/HLA-A/HLA-B/HLA-C/HLA-DMA/HLA-DQA1/HLA-DRB1/HLA-DRB5/HLA-E/HLA-F* | 10 |
| hsa05320 | Autoimmune thyroid disease | 1.16E-10 | *CD86/HLA-A/HLA-B/HLA-C/HLA-DMA/HLA-DQA1/HLA-DRB1/HLA-DRB5/HLA-E/HLA-F* | 10 |
| hsa05416 | Viral myocarditis | 3.46E-10 | *CD86/HLA-A/HLA-B/HLA-C/HLA-DMA/HLA-DQA1/HLA-DRB1/HLA-DRB5/HLA-E/HLA-F* | 10 |
| hsa04612 | Antigen processing and presentation | 4.34E-09 | *B2M/HLA-A/HLA-B/HLA-C/HLA-DMA/HLA-DQA1/HLA-DRB1/HLA-DRB5/HLA-E/HLA-F* | 10 |
| hsa04514 | Cell adhesion molecules | 1.71E-07 | *CD86/HLA-A/HLA-B/HLA-C/HLA-DMA/HLA-DQA1/HLA-DRB1/HLA-DRB5/HLA-E/HLA-F/PTPRC* | 11 |
| hsa04145 | Phagosome | 2.28E-06 | *C3/HLA-A/HLA-B/HLA-C/HLA-DMA/HLA-DQA1/HLA-DRB1/HLA-DRB5/HLA-E/HLA-F* | 10 |
| hsa05169 | Epstein-Barr virus infection | 3.10E-06 | *B2M/CXCL10/HLA-A/HLA-B/HLA-C/HLA-DMA/HLA-DQA1/HLA-DRB1/HLA-DRB5/HLA-E/HLA-F* | 11 |
| hsa05166 | Human T-cell leukemia virus 1 infection | 7.23E-06 | *B2M/HLA-A/HLA-B/HLA-C/HLA-DMA/HLA-DQA1/HLA-DRB1/HLA-DRB5/HLA-E/HLA-F/IL2RG* | 11 |
